# Toward Functional Oil Blends: Physicochemical and Nutritional Evaluation of Rapeseed–Hazelnut Oil Mixtures

**DOI:** 10.3390/foods14234008

**Published:** 2025-11-22

**Authors:** Marta Siol, Diana Mańko-Jurkowska, Izabela Stanaszek, Bartłomiej Zieniuk, Andrzej Bryś, Joanna Bryś

**Affiliations:** 1 Department of Chemistry, Institute of Food Sciences, Warsaw University of Life Sciences, 02-776 Warsaw, Poland; marta_siol@sggw.edu.pl (M.S.); bartlomiej_zieniuk@sggw.edu.pl (B.Z.); joanna_brys@sggw.edu.pl (J.B.); 2 Faculty of Food Technology, Warsaw University of Life Sciences, 02-776 Warsaw, Poland; stanaszekiza@gmail.com; 3 Department of Fundamentals of Engineering and Power Engineering, Institute of Mechanical Engineering, Warsaw University of Life Sciences, 02-787 Warsaw, Poland; andrzej_brys@sggw.edu.pl

**Keywords:** hazelnut oil, rapeseed oil, oil blend, fatty acid profile, health indices, oil quality, oxidative stability

## Abstract

The combination of rapeseed oil (RO) and hazelnut oil (HO) was selected to create a functional blend integrating two technologically complementary lipid matrices. RO is valued for its favorable fatty acid (FA) profile, particularly its low saturated FA content and the presence of essential polyunsaturated FAs, whereas HO is characterized by high monounsaturated FA levels and inherently greater resistance to oxidative deterioration. Blending these oils enables the formulation of mixtures that balance nutritional quality with improved physicochemical stability, without the need for chemical modification. Such an approach is relevant for applications requiring oils that retain desirable characteristics during storage and handling. In this context, the present study aimed to evaluate the quality characteristics, FA composition, triacylglycerol (TAG) structures, and oxidative stability of binary blends of RO and HO. Two commercial oils, as well as their blends in RO:HO volume ratios of 3:1, 1:1, and 1:3, were tested. The samples were stored under two temperature conditions: 4 °C (refrigeration) and 20 °C (room temperature), and analyzed after two and four months of storage. Initial MUFA content ranged from approx. 61–74%, increasing with HO proportion, whereas PUFA levels decreased accordingly (from ~28% in RO to ~10% in HO-rich blends). The *sn*-2 TAG position was predominantly occupied by unsaturated FAs (>80%). Statistical analysis (*p* < 0.05) showed that both storage time and temperature significantly affected PV, while no significant differences were observed in the overall proportions of SFA, MUFA, and PUFA. Blends with a higher proportion of RO exhibited increased AVs, suggesting greater susceptibility to hydrolytic changes, whereas mixtures enriched in HO demonstrated superior oxidative stability, as reflected by significantly lower peroxide values (*p* < 0.05), which can be attributed to their lower PUFA content. The 1RO:3HO blend exhibited the most favorable balance between beneficial nutritional indices and stability against quality deterioration over the storage period. The results indicated that the formulation of balanced mixtures combining the favorable FA profile of RO with the oxidative resistance of HO represents a promising approach for obtaining oils with improved functional and nutritional properties.

## 1. Introduction

Rapeseed oil (*Brassica napus* L.) is among the three most important vegetable oils worldwide, with its production having particular economic significance in Europe and China. Historically, its use was limited by the high content of erucic acid and glucosinolates; however, advances in plant breeding have led to the development of “double low” cultivars characterized by reduced levels of both compounds, enabling the broad utilization of rapeseed oil in human nutrition [1]. From a lipid profile perspective, rapeseed oil is distinguished by a favorable composition of fatty acids (FAs): oleic acid predominates, followed by linoleic and α-linolenic acids [2]. It also contains a low proportion of saturated FAs (approximately 7%) and offers an advantageous n-6/n-3 ratio of about 2:1 [3]. Rapeseed oil provides natural antioxidants, including tocopherols and phytosterols, which support oxidative stability and contribute to documented health effects such as improved lipid profile and reduced cardiovascular risk [4,5,6,7].

Hazelnut oil, commonly referred to as a fruit oil, is obtained from the kernels of *Corylus avellana* L., cultivated mainly in the Mediterranean region and parts of Central Europe [8,9]. The oil is characterized by a highly favorable FA profile, dominated by oleic acid (65–80%) with meaningful contributions from linoleic acid [8,10]. Hazelnut oil also contains significant amounts of α-tocopherol and phytosterols, particularly β-sitosterol [11,12], which enhance its oxidative stability. Owing to its mild nutty flavor and pleasant aroma, it is valued in high-quality culinary applications and is increasingly regarded as a functional oil due to its stable lipid matrix and natural antioxidant content.

Although both oils are well characterized individually, their lipid compositions are complementary. Rapeseed oil contributes essential polyunsaturated FAs and a low proportion of saturated FAs, while hazelnut oil provides a MUFA-rich matrix with inherently greater oxidative resistance. Blending such oils has been shown in previous studies—e.g., rapeseed–olive [13], rapeseed–sunflower [14], hazelnut skin oil–oleic palm [15], and sunflower–sesame systems [16]—to modulate FA profiles, enhance stability, and improve functional properties. However, systematic data on rapeseed–hazelnut blends remain scarce, particularly regarding medium-term storage behavior under typical household and refrigeration conditions. Moreover, the influence of blending ratio on the triacylglycerol (TAG) structure and oxidative stability of rapeseed–hazelnut mixtures has not been thoroughly examined.

Considering these complementary attributes and the limited scientific evidence on rapeseed–hazelnut oil mixtures, blending them represents a promising strategy for developing innovative functional formulations. In response to growing demands for improved nutritional quality and oxidative stability, oil blending offers a simple and cost-effective approach to tailor physicochemical and functional properties without chemical modification [17,18,19,20].

To address the identified research gap, the present study aimed to investigate the physicochemical stability and nutritional indices of rapeseed–hazelnut oil blends stored under room temperature (20 °C) and refrigeration (4 °C), analyzed after two and four months. This work elucidates how blending ratios affect FA composition, TAG structure, and oxidative stability, providing insights for the formulation of nutritionally balanced and oxidation-resistant edible oils.

## 2. Materials and Methods

### 2.1. Materials

Two commercial oils—rapeseed oil (RO) and hazelnut oil (HO)—as well as their blends in volume ratios of 3:1, 1:1, and 1:3 (RO:HO), were tested in this study. The oils were purchased from the same producer, within their declared shelf life, and tested before the expiration date. According to the manufacturer, the oils were cold-pressed, unrefined, unfiltered, and 100% natural, with no added antioxidants. They were produced from raw materials cultivated in Poland and supplied in dark glass bottles.

Blends were prepared by volumetrically combining RO and HO in the target RO:HO ratios in amber borosilicate glassware and mixing by gentle magnetic stirring at ambient laboratory temperature until optically homogeneous. To minimize oxidative artifacts, mixing was conducted under low-light conditions with minimal headspace, after which the blends were immediately aliquoted into dark-glass vials and tightly capped. Initial determinations were performed on both the individual oils and the freshly prepared blends.

The oils were stored at 4 ± 1 °C, following the manufacturer’s recommendations, as well as at 20 ± 2 °C (room temperature). Subsequent analyses were conducted after two and four months of storage, during which all samples were protected from light and oxygen.

All solvents and reagents used were of chromatographic or analytical grade, sourced from Avantor (Gliwice, Poland), except for standard compounds, which were supplied by Sigma–Aldrich (Saint Louis, MO, USA).

### 2.2. Methods

#### 2.2.1. Determination of Fatty Acid Composition by Gas Chromatography

The fatty acid composition of each oil sample was determined using gas chromatography, employing a YL6100 GC Clarity system (Young Lin Bldg., Anyang, Hogye-dong, Korea) equipped with a flame ionization detector (FID) and a BPX-70 capillary column (SGE Analytical Science, Milton Keynes, UK). Fatty acid methyl esters (FAMEs) were prepared in accordance with the EN ISO 5509:2001 standard [21]. Nitrogen was used as the carrier gas at a constant flow of 1.0 mL·min^−1^ (constant-flow mode). Injections were made in split mode with a split ratio of 1:50. The oven program was: 70 °C for 0.5 min; ramp at 15 °C·min^−1^ to 160 °C; ramp at 1.1 °C·min^−1^ to 200 °C (isothermal hold 12 min); ramp at 30 °C·min^−1^ to 225 °C (hold 1 min). Injector and detector temperatures were set to 225 °C and 250 °C, respectively. Individual FAs were identified by comparing their retention times with those of a reference standard mixture (Supelco 37 Component FAME Mix, Sigma-Aldrich, Bellefonte, PA, USA). The relative content of each FA was expressed as a percentage of the total amount of identified FAs.

#### 2.2.2. Health-Related Lipid Indices

The FA composition was used to calculate several health-related lipid indices for the tested oils and their blends. The atherogenic index (AI) and thrombogenic index (TI) were calculated using the formulas proposed by Ulbricht and Southgate (Equations (1) and (2)) [22], while the hypocholesterolemic/hypercholesterolemic ratio (h/H) was determined according to Equation (3) [23]:

(1) $$AI=\frac{C12:0+\left(4\times C14:0\right)+C16:0}{\sum MUFA+\sum PUFAn-6+\sum PUFAn-3}$$

(2) $$TI=\frac{C14:0+C16:0+C18:0}{0.5\times \sum MUFA+\left(0.5\times \sum PUFAn-6\right)+\left(3\times \sum PUFAn-3\right)+\frac{\sum PUFAn-3}{\sum PUFAn-6}}$$

(3) $$h/H=\frac{cis-C18:1+\sum PUFA}{C12:0+C14:0+C16:0}$$

where FA, fatty acids; UFA, unsaturated fatty acids; SFA, saturated fatty acids; MUFA, monounsaturated fatty acids; PUFA, polyunsaturated fatty acids.

#### 2.2.3. Distribution of Fatty Acids in Triacylglycerols Using Enzymatic Hydrolysis

The enzymatic hydrolysis method described by Bandara et al. [24] was used to determine the structure of triacylglycerols (TAGs) of the tested samples. Based on the compositions of the isolated *sn*-2 monoacylglycerols (MAGs) and the starting TAGs, the composition of the FAs in the *sn*-1,3 positions was determined (Equations (4) and (5)):

(4) $$sn-2=\;\frac{{(FA}_{insn-2MAG})}{3\times {(FA}_{inTAG})}\times 100\%$$

(5) $$sn-1,3=\frac{3\times {(FA}_{inTAG})-({FA}_{insn-2MAG})}{2}$$

where *sn*-1,3, the content of a given fatty acid in *sn*-1 and *sn*-3 positions [%]; FA in TAG, the content of a given fatty acid in the starting triacylglycerols [%]; FA in *sn*-2 MAG, the content of a given fatty acid in *sn*-2 monoacylglycerols [%].

#### 2.2.4. Determination of Acid and Peroxide Values by Potentiometric Titration Method

The physicochemical quality of the tested oils and their mixtures was assessed based on standard lipid quality parameters. The acid value (AV), which indicates the extent of hydrolytic degradation, was determined following the AOCS Official Method Te 1a-64 [25]. The peroxide value (PV), reflecting the concentration of primary oxidation products, was measured in accordance with AOCS Cd 8b-90 [26]. Both AV and PV were determined using an automatic titrator (TitraLab AT1000 Series, Hach Lange, Wroclaw, Poland).

#### 2.2.5. Determination of Oxidative Stability Using Pressure Differential Scanning Calorimetry

Pressure differential scanning calorimetry (PDSC) was employed to assess the oxidative stability of the tested oils and their mixtures using a DSC Q20P thermal analyzer (TA Instruments, New Castle, DE, USA). Oil samples (3.0–4.0 mg) were weighed and placed in open aluminum pans within the sample chamber of the PDSC cell. Each measurement was performed with a single oil sample and an empty reference pan. Measurements were carried out under an oxygen atmosphere at an initial pressure of approximately 1400 kPa. Each analysis was conducted isothermally at 120 °C. The resulting PDSC curves were analyzed using Universal Analysis 2000 software. The oxidation time (τ_max_) was determined based on the point of maximum heat flow rate, with a measurement accuracy of ±0.005.

### 2.3. Statistical Analysis

All experimental samples were prepared according to a factorial arrangement including oil formulation (RO, HO, and RO:HO blends at 3:1, 1:1, and 1:3), storage temperature (4 °C and 20 °C), and storage time (0, 2, and 4 months), yielding 30 formulation × temperature × time combinations. For each combination, samples were analyzed in triplicate, and the results are expressed as mean ± standard deviation (SD). Statistical analysis was performed using Statistica software, version 13.3 (TIBCO Software Inc., Palo Alto, CA, USA). Differences between means were evaluated using two-way analysis of variance (ANOVA), followed by Tukey’s post hoc test. Statistical significance was considered at *p* ≤ 0.05. In addition to univariate tests, multivariate analyses were conducted to explore relationships among variables and to characterize the samples. Since fatty-acid classes are compositional, SFA, MUFA, and PUFA were first transformed into isometric log-ratio (ilr) coordinates, calculated as (Equations (6) and (7)):

(6) $$ilr1=\sqrt{\frac{1}{2}}\times ln\left(\frac{MUFA}{SFA}\right)$$

(7) $$ilr2=\sqrt{\frac{2}{3}}\times \left(\frac{lnMUFA+lnSFA}{2}-lnPUFA\right)$$

The multivariate data matrix for both PCA and HCA included ilr1, ilr2, n-6/n-3, AI, TI, h/H, PV, AV, and τ_max_. All variables used in the multivariate analyses were z-standardized. Principal component analysis (PCA) was used to summarize covariation and visualize sample separation by formulation and storage. Hierarchical cluster analysis (HCA) was conducted on the same standardized variables using Ward’s method with Euclidean distance. Pearson correlation analysis (shown as a heatmap) was also performed on the ilr-based composition along with the non-compositional variables listed above.

## 3. Results and Discussion

### 3.1. Fatty Acid Profile

Edible oils and fats of plant origin are complex biological systems composed primarily of glycerol esters with different fatty acid (FA) chains [27]. Their physical and chemical properties are strongly dependent on the type and ratio of FAs incorporated into triacylglycerol (TAG) molecules [28]. Variations in chain length, degree of unsaturation, and spatial configuration of FAs significantly affect melting behavior, oxidative stability, and nutritional quality, making the FA composition a key determinant of oil functionality in both food and industrial applications. In the case of oil blends, the proportions of saturated (SFA), monounsaturated (MUFA), and polyunsaturated fatty acids (PUFA) can be adjusted, which allows for the optimization of both nutritional profile and technological properties of the final product.

**Figure 1:**
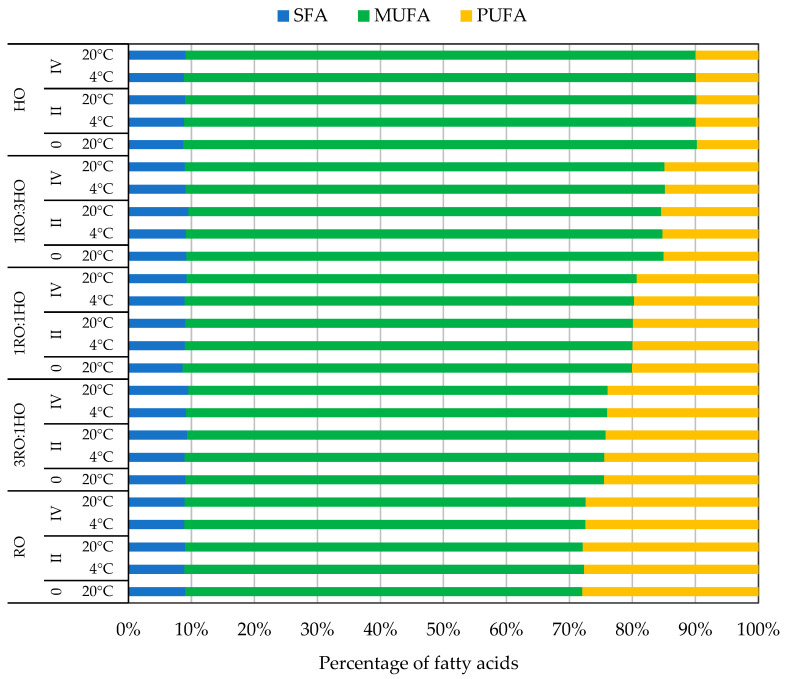
The content of saturated (SFA), monounsaturated (MUFA) and polyunsaturated fatty acids (PUFA) for tested oils and their blends, where RO, rapeseed oil; HO, hazelnut oil; 3RO:1HO, 1RO:1HO, and 1RO:3HO—3:1, 1:1, and 1:3 (*v*/*v*) rapeseed oil to hazelnut oil blends, respectively, immediately after opening (0) and after two (II) and four months (IV) of storage at 4 °C and 20 °C. Average proportions of SFA, MUFA, and PUFA calculated based on the mean fatty acid composition (*n* = 3). The detailed FA data used for these calculations are presented in the Supplementary Materials.

As shown in Figure 1, the FA composition of HO was clearly dominated by MUFAs, which represented the vast majority of total FAs, primarily due to the high oleic acid content (~81%; Tables S1 and S2, Supplementary Materials). PUFAs occurred at much lower levels, with linoleic acid forming the second most abundant fraction (above 9%; Table S1). In contrast, RO exhibited a more balanced profile, characterized by a substantially higher proportion of PUFAs, including both linoleic (~19%) and α-linolenic acids (~9%), accompanied by notable amounts of oleic acid (~62%; Table S1). SFAa were present in relatively low amounts in both oils (~9%; Table S1).

These findings are in line with earlier literature reports. For example, Zambiazi et al. [29] reported a MUFA content of 62.61% in rapessed oil, which closely corresponds to the value obtained in the present study (~63%; Table S1). When comparing MUFA levels across different vegetable oils, rapeseed oil can be classified among oils with relatively high MUFA content, although even higher values have been reported for sunflower, soybean, and corn oils. Hazelnut oil, in turn, is considered one of the richest sources of MUFA, with contents ranging from 75.10% to 82.10% [28], which closely aligns with the results obtained in this study (~82%; Table S1). Literature data further indicate that the overall FA profile of hazelnut oil is highly similar to that of olive oil [30], particularly regarding its elevated oleic acid content.

In the tested oil blends, the relative contributions of MUFA and PUFA varied according to the mixing ratio: increasing the proportion of HO raised the MUFA content, whereas higher levels of RO enhanced the PUFA fraction (Figure 1).

Based on the statistical analysis of individual FAs (full data provided in the Supplementary Materials), no significant differences were detected in the overall proportions of SFA, MUFA, and PUFA between storage temperatures (*p* > 0.05). Because the statistical tests were conducted on individual FAs and not on grouped FA categories, the averaged SFA/MUFA/PUFA values shown in Figure 1 are intended only to visualize general compositional trends rather than statistical significance. In contrast, storage time influenced the relative abundance of selected individual FAs, which in some cases resulted in measurable shifts within the broader FA classes. For example, a statistically significant increase in MUFA content was observed in the sample containing 75% (*v*/*v*) RO (3RO:1HO) after four months at 20 °C, as well as in RO stored under refrigeration for two and four months, compared with their initial values. Conversely, HO samples stored at 20 °C for two and four months showed a statistically significant decrease in MUFA content, whereas blends with equal proportions of both oils (1RO:1HO) and those enriched in HO (1RO:3HO) exhibited no significant changes over the same period. These results demonstrate that storage time and blend composition influence the stability of individual FAs, while temperature exerts only a minor effect under the tested conditions.

Evaluation of the FA profile (Tables S1 and S2, Supplementary Materials) of tested oils and their blends enables the calculation of several indices that are commonly used to predict the potential nutritional and health effects of dietary fats (Table 1).

Health-related lipid indices, particularly the atherogenic index (AI) and thrombogenic index (TI), are among the most reliable and widely applied markers for assessing the impact of FAs on cardiovascular disease risk. The AI provides an early indication of atherosclerosis development and is associated with multiple inflammatory pathways, while the TI reflects the tendency for clot formation in blood vessels, both of which are directly linked to cardiovascular health [31]. For oils intended for human consumption, values of AI < 1.0 and TI < 0.5 are considered desirable, together with a high hypocholesterolemic/hypercholesterolemic ratio (h/H) [22].

In the present study, all analyzed oils and their blends met these criteria, with AI ranging from 0.06 to 0.07 and TI from 0.12 to 0.19 (Table 1). These results indicate a very low atherogenic and thrombogenic potential. Importantly, no significant changes in these indices were observed during storage, regardless of temperature or time. Recent literature demonstrates that AI is increasingly applied to a wide range of food matrices—not only oils but also more structurally complex products. For instance, in the study by Alam et al. (2025) [32], the lipid fraction of meat analog formulations showed AI values of ~0.39–0.40, despite being plant-based. Such differences primarily reflect the distinct lipid matrices and susceptibility to oxidation: while pure edible oils contain well-defined FA profiles with low SFA content, meat analogs (and similar composite foods) typically include protein–lipid interactions, processing-derived volatiles, and structural components that promote oxidative degradation of MUFA and PUFA. As a result, their AI values are markedly higher.

The h/H ratios of tested oils and their blends were consistently high (>14.39), further supporting the health-promoting potential. The highest h/H values were observed for RO (up to 17.38) and for blends with a predominance of this oil, reflecting its beneficial hypocholesterolemic effect. Conversely, the lowest h/H values were noted for blends rich in HO, particularly 1RO:3HO (13.97–14.39), which may indicate a less beneficial impact on lipid metabolism. Storage time and temperature exerted only minor effects on h/H values.

In addition to these indices, the ratio of omega-6 to omega-3 FAs (n-6/n-3) was also evaluated, as it represents a key determinant of dietary fat quality and plays a crucial role in maintaining metabolic balance and preventing the development of chronic diseases. RO exhibited a well-balanced n-6/n-3 ratio of 2.09, consistent with previously reported data [33]. This value falls within the range recommended by international health authorities, including the World Health Organization (WHO) and the Food and Agriculture Organization (FAO), which indicate that an n-6/n-3 ratio between 1:1 and 5:1 is generally considered favorable for cardiovascular and metabolic health [34,35]. By contrast, HO was characterized by a markedly high n-6/n-3 ratio of 56.41, far exceeding the recommended values. Such an imbalance is considered nutritionally unfavorable, as an excessive predominance of omega-6 over omega-3 may promote inflammatory processes and increase the risk of cardiovascular disease. An increase in the proportion of HO within the blends led to a reduction in both omega-3 and omega-6 levels (Tables S1 and S2), ultimately resulting in an elevated n-6/n-3 ratio (Table 1). Importantly, in all tested blends, the ratios remained within the range recommended by international health authorities. This consistent balance underscores the favorable nutritional quality of rapeseed–hazelnut oil blends, suggesting that their inclusion in the human diet may provide meaningful health benefits through an optimized intake of essential FAs.

The comparison of health-related lipid indices between our results and those reported by Khalili Tilami and Kouřimská [31] revealed a high degree of consistency. For rapeseed oil, both studies indicated very low AI values (0.05 vs. 0.06) and similarly low TI values (0.09 vs. 0.12), while for hazelnut oil, identical AI values (0.06) and comparable TI values (0.16 vs. 0.18) were observed. Likewise, the n-6/n-3 ratio for RO in our work (2.09) was in close agreement with the literature (~1.89), whereas HO showed extremely high values in both datasets (56.41 vs. 50.00), highlighting the predominance of omega-6 FAs over omega-3.

Altogether, RO and tested blends with their high proportion were characterized by the most favorable nutritional indices (low AI and TI, high h/H, and low n-6/n-3 ratio), making them a better dietary choice for cardiovascular disease prevention. HO, despite its high content of MUFAs, showed an unfavorable n-6/n-3 ratio, which limits its nutritional value as a stand-alone dietary component.

### 3.2. Fatty Acid Distribution

The positional distribution of FAs within TAGs markedly affects lipid digestion and bioavailability. Unsaturated and medium-chain FAs located at the internal (*sn*-2) position are preferentially absorbed, whereas long-chain SFAs released from the external (*sn*-1 and *sn*-3) positions may form insoluble calcium or magnesium soaps in the intestinal lumen, thereby limiting their absorption [36].

In plants and animals, specific FAs occupy characteristic positions within the TAG molecule due to the enzymatic specificity during biosynthesis. In vegetable oils, SFAs are mainly found at the *sn*-1/*sn*-3 positions, while unsaturated FAs (MUFA and PUFA) are typically found at the *sn*-2 position; in animal fats, SFAs are predominantly located at the *sn*-2 position, enhancing their digestibility. However, the higher overall proportion of unsaturated fatty acids in plant TAGs facilitates easier total fat absorption and exerts beneficial effects on lipid metabolism and cardiovascular health. Thus, both the chemical composition and positional distribution of FAs within TAG molecules are key determinants of fat digestibility, bioavailability, and physiological impact [36,37].

**Figure 2:**
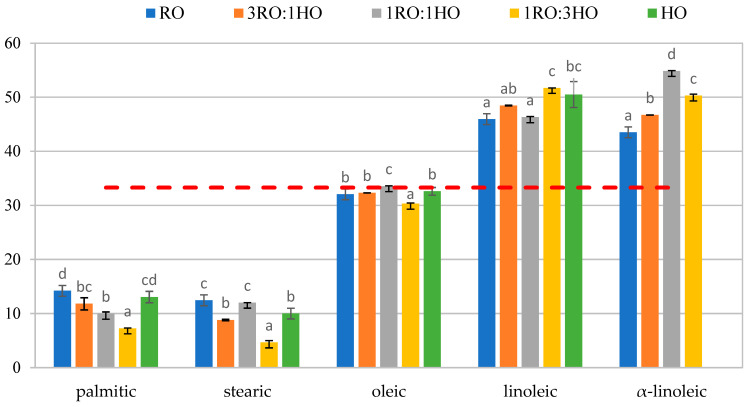
The percentage of a given fatty acid in the *sn*-2 position of triacylglycerols (TAGs) (n = 3) of tested oils and their mixtures, where RO, rapeseed oil; HO, hazelnut oil; 3RO:1HO, 1RO:1HO, and 1RO:3HO—3:1, 1:1, and 1:3 (*v*/*v*) rapeseed oil to hazelnut oil blends, respectively. The dotted line indicates the statistical (even) distribution of FA between three TAG positions (33%). Data represent mean values (n = 3). The different letters indicate significantly different values (*p* ≤ 0.05).

Figure 2 presents the percentage share of individual FAs in the *sn*-2 position of TAGs. The results confirmed that in the studied mixtures, SFAs predominantly occupied the outer *sn*-1 and *sn*-3 positions of TAGs. Both palmitic and stearic acids followed this trend, showing the lowest proportions in the *sn*-2 position in RO and the highest in the 75% RO mixture (3RO:1HO). Specifically, palmitic acid accounted for 7.28% and 14.18% of the *sn*-2 position in RO and 3RO:1HO, respectively, whereas stearic acid contributed 4.65% and 12.44% in the same samples.

The distribution of MUFAs in TAGs was relatively even across all positions. Among the major MUFAs present in the *sn*-2 position, oleic acid was dominant, with its proportion ranging from 30.32% in RO to 33.54% in the mixture containing 75% hazelnut oil (1RO:3HO). In contrast to SFAs, which were mainly located at the outer positions, PUFAs were predominantly incorporated into the inner *sn*-2 position of TAGs. The major PUFAs identified in this position were linoleic and α-linolenic acids. Linoleic acid accounted for 46.31% in the mixture with the lowest RO content (1RO:3HO) and 51.68% in pure RO. α-linolenic acid ranged from 43.48% in 3RO:1HO to 54.88% in 1RO:3HO, indicating that increasing the share of RO in the blend reduced the proportion of α-linolenic acid incorporated into the *sn*-2 position. These findings are consistent with previously reported data in the literature. This stereospecific arrangement has important implications for the functionality and nutritional quality of the analyzed mixtures. The predominance of long-chain SFAs in the outer *sn*-1 and *sn*-3 positions may limit their bioavailability due to the formation of insoluble calcium and magnesium soaps, which not only reduces fat absorption efficiency but also leads to losses of these minerals in the intestinal lumen, particularly in blends with a lower proportion of RO [38]. In contrast, the presence of MUFAs and PUFAs in the *sn*-2 position favors more efficient digestion, as pancreatic lipase leaves FAs in this position as 2-monoacylglycerols, which are preferentially and effectively absorbed in the small intestine [36]. Consequently, oils enriched in unsaturated FAs at the *sn*-2 position exhibit a more favorable nutritional profile and higher lipid bioavailability.

RO is rich in unsaturated FAs, mainly oleic acid (C18:1), linoleic acid (C18:2), and linolenic acid (C18:3), while the proportion of the saturated palmitic acid (C16:0) is low. According to Schmidt et al. [39], approximately 0.6 mol % of palmitic acid, 48.0 mol % of oleic acid, 37.8 mol % of linoleic acid, and 13.5 mol % of linolenic acid are located in the *sn*-2 position, whereas in the *sn*-1,3 positions these values are 7.8 mol %, 61.4 mol %, 17.6 mol %, and 9.4 mol %, respectively. Consequently, in RO, SFAs preferentially occupy the outer positions, while PUFAs are concentrated in the inner position.

According to Ciemniewska-Żytkiewicz et al. [38], in HO, the distribution of FAs within TAG molecules depends on their degree of unsaturation. Oleic acid, the major FA in HO, is relatively evenly distributed among the internal and external positions, whereas linoleic acid is mainly located at the *sn*-2 position. In contrast, SFAs, particularly palmitic acid, are predominantly found at the outer *sn*-1 and *sn*-3 positions. Although the enrichment of the *sn*-2 position with unsaturated FAs is generally considered beneficial, the presence of palmitic acid at the outer positions is less favorable. During digestion, pancreatic lipase hydrolyzes FAs at the *sn*-1 and *sn*-3 positions, releasing free fatty acids that can form insoluble calcium soaps in the intestine. This reduces the intestinal absorption of both fatty acids and calcium, thereby diminishing the nutritional value of oils rich in palmitic acid located in external TAG positions.

### 3.3. Evaluation of Oil Quality

Cold-pressed oils, especially those rich in PUFAs, as well as their blends, are highly susceptible to oxidative deterioration during storage. Oxidative processes not only affect the nutritional properties of oils but also determine their overall quality [40]. In this study, the oils and their blends were stored in dark glass bottles under two conditions—refrigeration (4 ± 1 °C) and room temperature (20 ± 2 °C)—for up to four months, reflecting the most common storage practices in households, where oils are typically kept either at ambient temperature or under refrigeration according to the manufacturer’s recommendations. To evaluate oil quality under these conditions, PV, AV, and oxidative stability were determined. Such an approach not only provides laboratory-based assessment of degradation pathways but also mirrors real-world consumer behavior. Consequently, the findings offer practical insights into how storage choices influence the functional shelf-life of specialty oils, informing both users and producers about conditions that best preserve their nutritional and sensory attributes.

#### 3.3.1. The Acid and Peroxide Values

AV and PV are two fundamental analytical parameters applied in the assessment of the quality and freshness of vegetable oils. The AV expresses the degree of hydrolytic degradation of TAGs and is defined as the quantity of potassium hydroxide (mg KOH) required to neutralize the free fatty acids (FFAs) contained in one gram of oil. It is a key indicator of freshness and hydrolytic stability, as elevated AVs correspond to increased FFA concentrations, which are more prone to autoxidation and, consequently, can accelerate oxidative [41,42,43]. In accordance with the Codex Alimentarius, the AV of cold-pressed vegetable oils should not exceed 4 mg KOH/g fat [44].

The PV, on the other hand, quantifies the concentration of hydroperoxides, which represent the primary products of lipid oxidation. It therefore provides a direct measure of the progression of oxidative processes and serves as an indicator of oil freshness, with higher PVs, reflecting an advanced stage of rancidity [45,46]. The rate of oxidation is influenced by factors such as the presence of oxygen, elevated temperature, exposure to light, and catalytic activity of transition metals, whereas susceptibility depends on the FA composition, TAG structure, and the presence of pro-oxidants or natural antioxidants [42,45,47]. For cold-pressed vegetable oils, the Codex Alimentarius specifies a maximum permissible PV of 15 meq O_2_/kg fat [44]. The average values of the AV and PV of the tested oils and their mixtures are summarized in Table 2.

Immediately after opening, the highest AV was observed for RO (5.81 ± 0.01 mg KOH/g), whereas the lowest was recorded for HO (1.98 ± 0.03 mg KOH/g). Intermediate levels were determined for the blends: 3RO:1HO—4.98 ± 0.21, 1RO:1HO—4.00 ± 0.01, and 1RO:3HO—3.30 ± 0.04 mg KOH/g. These results indicate that the incorporation of HO into mixtures improved hydrolytic stability, as reflected by reduced AVs.

During storage, all samples exhibited a gradual increase in AV, with more pronounced changes at 20 °C compared to 4 °C. The temperature effect was statistically significant within each formulation (20 °C > 4 °C after four months), except for HO at 4 °C, where the change vs. time 0 was not significant (*p* > 0.05). In RO, values after 4 months reached 5.86 ± 0.05 (4 °C) and 6.21 ± 0.27 (20 °C), consistently exceeding the Codex Alimentarius threshold (4 mg KOH/g). Similarly, in 3RO:1HO, AV remained elevated (5.20 ± 0.03–5.29 ± 0.11), confirming that blends dominated by RO display limited hydrolytic stability. The 1RO:1HO blend also exceeded the recommended value after 4 months (4.48 ± 0.24 at 20 °C), with a clear temperature dependence, and the increase at 20 °C was significant relative to baseline, while 1RO:3HO exhibited the lowest values among blends (3.17 ± 0.01–3.23 ± 0.02), remaining below the Codex limit throughout storage. A small but significant rise in AV was detected for 1RO:3HO at 20 °C by month 4, whereas the change at 4 °C was not significant. HO itself demonstrated the highest hydrolytic stability, showing only a slight increase over 4 months (1.98 ± 0.03 → 2.31 ± 0.09), irrespective of temperature. This increase was significant at 20 °C but not at 4 °C (*p* > 0.05).

Such discrepancies are largely a consequence of differences in the FA composition of the oils. RO, characterized by a higher content of PUFAs, was more susceptible to hydrolysis, explaining its consistently elevated AV. In contrast, HO, rich in MUFAs and with a lower proportion of PUFAs, exhibited markedly greater stability, since MUFAs are less prone to hydrolytic cleavage. The protective effect of HO in blends was particularly evident in 1RO:3HO, where the predominance of MUFAs effectively limited the increase in AV during storage.

In the study by Szydłowska-Czerniak et al. [48], the AV of commercial refined rapeseed oils ranged from 1.02 to 3.21 mg KOH/g, which was considerably lower than the values obtained in the present study. Similarly, Zheng et al. [49] reported lower AVs for rapeseed oil, both immediately after extraction (0.52 mg KOH/g) and after two months of storage (0.89 mg KOH/g). Cold-pressed walnut oils were also characterized by markedly lower AVs, both immediately after pressing and even after nine months of storage [50]. The differences between the AVs determined in the present study and those reported in the literature may primarily be attributed to the specificity of the analyzed samples. In the cited studies [48,49], commercial refined oils derived from different batches of raw material processed under different technological conditions were evaluated, which could have had a significant impact on the content of FFAs. Another important factor is the quality of the raw material used for oil production—seeds may differ in moisture content, susceptibility to mechanical damage, and the degree of enzymatic activity, all of which directly affect acid values. Furthermore, storage conditions of commercial oils before purchase and analysis (time, temperature, light exposure) may have promoted progressive TAG hydrolysis, which explains the higher AVs observed in the present study. Consistently, our statistical analysis supports that technological and raw-material factors are associated with significantly different AV levels across oils and blends (*p* < 0.05).

In addition to hydrolytic stability, the oxidative stability of the oils was evaluated by determining the PV. Immediately after opening, all samples exhibited low PVs, well below the Codex Alimentarius limit of 15 meq O_2_/kg. The highest initial value was observed for RO (6.21 ± 0.14 meq O_2_/kg), while the lowest was recorded for HO (1.98 ± 0.01 meq O_2_/kg), with blends ranging between these extremes (e.g., 3RO:1HO—4.84 ± 0.05, 1RO:1HO—3.85 ± 0.49, 1RO:3HO—2.56 ± 0.11 meq O_2_/kg).

After 2 months of storage at 4 °C, only minor increases in PV were detected, generally within 0.2–0.4 units, indicating effective inhibition of autoxidation under refrigeration. At 20 °C, however, changes were more pronounced, particularly in RO, where PV rose to 11.06 ± 0.59 after 2 months, nearly doubling compared to the initial level. Both the time effect (0 → 2 months → 4 months) and the temperature effect (20 °C vs. 4 °C) were significant for RO. In contrast, HO and the 1RO:3HO blend maintained stable values, not exceeding 3.6 meq O_2_/kg. For these two, several comparisons between 20 °C and 4 °C remained nonsignificant.

After 4 months, the effect of storage temperature became more evident. At 4 °C, all samples remained within the Codex limit, with PV increases remaining moderate (e.g., RO—8.46 ± 0.21, HO—2.87 ± 0.04 meq O_2_/kg). By contrast, at 20 °C, RO (22.45 ± 4.15) and 3RO:1HO (21.66 ± 2.79) exhibited a sharp accumulation of hydroperoxides, exceeding the permissible limit several times. Conversely, HO (3.56 ± 0.21) and 1RO:3HO (3.70 ± 0.28) showed only slight increases, remaining well below the threshold. Their final PVs did not differ significantly from refrigerated counterparts in several pairwise comparisons.

In the studies by Szydłowska-Czerniak et al. [48], the PVs for commercial refined rapeseed oils ranged from 0.04 to 2.04 meq O_2_/kg, which was significantly lower than the values obtained in the rapeseed oil analyzed in this study. In turn, Konuskan et al. [51] reported a PV of 9.46 meq O_2_/kg for cold-pressed rapeseed oil, which exceeded the result obtained in the RO tested immediately after opening the package. In the case of HO, the PVs determined in this study were higher than those reported by Ciemniewska-Żytkiewicz et al. [52]. A similar trend was observed in the study by Ayyildiz et al. [53], where the PV for hazelnut oil was 0.51 meq O_2_/kg, which is significantly lower than in this analysis. Data on oil blends, especially those with varying proportions of RO and HO, are very limited. The available studies lack detailed information on the effect of the proportions of both oils on changes in AV and PV during storage. The findings of this study provide novel insights into the influence of FA composition on the storage stability of vegetable oils. Statistical analysis supports these conclusions, indicating significant effects of time, temperature, and composition on PV (*p* < 0.05).

Overall, the results demonstrate that both AV and PV are governed not only by storage time and temperature but also by the relative proportion of MUFAs and PUFAs in the oil matrix. Samples with a high RO content (RO, 3RO:1HO) exhibited the lowest hydrolytic and oxidative stability, whereas HO and blends with its predominance (1RO:3HO) maintained both AV and PV within acceptable limits. These findings confirm the protective role of MUFA-rich oils, particularly HO, in mitigating hydrolytic and oxidative deterioration during storage.

#### 3.3.2. Evaluation of Oxidative Stability

Lipid oxidation is one of the key processes affecting the quality of edible oils. It directly determines their nutritional properties, sensory attributes, and storage stability, as faster oxidation shortens shelf life and accelerates quality deterioration [41]. Due to the relatively slow oxidation rate of oils under ambient conditions, accelerated oxidation tests are increasingly used in research. These methods allow the prediction of real-time oxidative stability while requiring small sample amounts, short analysis times, and no chemical reagents. Among them, pressure differential scanning calorimetry (PDSC) is commonly applied for edible oils. In this technique, the oxygen concentration is adjusted to exceed that at ambient pressure, thereby expediting the oxidation process [54]. The resulting oxidation time (τ_max_) represents the time required to reach the maximum exothermic signal on the PDSC curve, corresponding to the most extensive oxidative changes in the sample (maximum rate of heat flow) [55]. In the present study, PDSC was employed to evaluate the oxidative stability of RO and HO blends differing in composition and storage conditions, providing a reliable comparison of their thermal oxidative behavior.

Before storage, the shortest τ_max_, and thus the lowest oxidative stability, was observed for RO (62.61 ± 2.22 min), whereas the longest τ_max_ was recorded for HO (160.32 ± 3.84 min). The markedly shorter τ_max_ of RO compared with HO can be attributed to differences in their FA composition. RO contained substantially higher proportions of linoleic acid (18.91%) and α-linolenic acid (8.70%) than HO (9.59% and 0.14%, respectively). As the autooxidation rates of oleic, linoleic, and linolenic acids are approximately 1:40:100, respectively [56], the higher content of PUFAs in RO directly explains its lower oxidative stability.

The τ_max_ values obtained in this study are consistent with those previously reported for refined rapeseed oil analyzed under comparable PDSC conditions by Symoniuk et al. [54] (66.61–74.78 min) and [57] (60.28–67.05 min). However, the oils examined in the former study [54] contained a slightly higher percentage of linoleic acid (17.28–17.59%) and a lower percentage of oleic acid (63.33–64.56%) compared with the RO analyzed in the present study (18.91 and 61.55%, respectively). In contrast, the τ_max_ values obtained for HO fell within the broad range previously reported at 120 °C by Ciemniewska-Żytkiewicz et al. [55] (119.25–191.06 min), confirming the inherently higher oxidative stability of nut oils rich in MUFAs.

The oxidative stability of the RO:HO blends depended strongly on the proportion of HO in the mixture, increasing with its higher content. The lowest stability was observed for the 3RO:1HO blend (τ_max_ = 69.32 ± 0.83 min), whereas the highest value was recorded for the 1RO:3HO blend (τ_max_ = 115.31 ± 1.10 min). The oxidation behavior of the blends also varied depending on the stored conditions. Literature data indicate that oxidation time generally decreases during storage due to progressive oxidative degradation, particularly at room temperature [58,59]. This trend was clearly visible for RO, which exhibited the longest τ_max_ immediately after opening and a gradual decline over time—from 51.58 ± 1.32 min after 2 months to 35.99 ± 0.01 min after 4 months at 20 °C.

The average values of τ_max_ of the tested oils and their blends are presented in Table 3.

However, different patterns were observed for HO and its blends with RO. In the case of HO, the shortest τ_max_ was observed immediately after opening, while it increased with prolonged storage, although the differences were no statistically significant with respect to temperature. For the 1:1 (*v*/*v*) RO:HO blend, storage time and temperature exerted the least effect on τ_max_, with no statistically significant differences observed, except for a notable increase after four months at 4 °C. A similar tendency was found for the 1RO:3HO (*v*/*v*) mixture. Conversely, the greatest discrepancies among results were noted for the 3RO:1HO (*v*/*v*) blend, indicating the strongest influence of both storage temperature and storage duration. In this blend, a pronounced decrease in τ_max_ was observed after 4 months at 20 °C, whereas at 4 °C, the value markedly increased, reaching its maximum after the same storage period.

These findings clearly demonstrate the combined impact of both oils on the oxidative stability of their mixtures, highlighting the complex interactions between the components that may result in either synergistic or antagonistic effects depending on their proportion and storage conditions. In the context of existing stabilization strategies for edible oils, which frequently rely on the addition of natural or synthetic antioxidants (such as tocopherols, plant extracts, or phenolic-rich formulations), the present blend-based approach offers a formulation-driven alternative. The literature reports that typical antioxidant additives may extend induction times by approximately 20–60%, depending on the compound, concentration, and oil matrix [60,61]. In the present study, comparable improvements in oxidative stability were achieved solely by adjusting the RO–HO ratio, with HO-rich blends exhibiting markedly prolonged τ_max_ values without the need for supplementation. Although a direct experimental comparison with antioxidant-enriched oils was beyond the scope of this work, these findings demonstrate that modulating the MUFA/PUFA balance through blending can serve as an effective clean-label strategy for enhancing oxidative stability, complementary to more conventional antioxidant-based approaches.

The observed differences in the oxidative stability of RO, HO, and their blends during storage depend not only on their FA composition. In contrast to refined oils, cold-pressed oils often exhibit a higher initial degree of autooxidation and less predictable oxidative behavior, as their stability is strongly influenced by naturally occurring compounds with both antioxidant and prooxidant activity [62,63]. According to the literature, RO contains considerable amounts of tocopherols (mainly γ-tocopherol), phenolics, carotenoids, and phytosterols, which can contribute to antioxidative protection, whereas chlorophylls and trace metals may promote oxidation [2,64,65]. In contrast, HO typically contains lower levels of total tocopherols (with α-tocopherol as the dominant homolog) but relatively high amounts of total phenolic compounds and pigments, which may act as antioxidants or, under certain conditions, exhibit prooxidant behavior [50,66].

In oil blends, these constituents can interact in complex ways, potentially enhancing or diminishing oxidative stability through synergistic or antagonistic effects. The balance between these opposing factors plays a decisive role in determining the overall oxidative behavior of the blends [67]. A comprehensive assessment of these mechanisms was beyond the scope of the present study and warrants further investigation.

An especially noteworthy observation was the increase in the oxidative stability of HO during storage. These phenomena may result from the gradual activation or enhanced effectiveness of natural antioxidants, such as phenolic compounds and pigments, which can exhibit increased reactivity under storage conditions. Previous studies have indicated that phenolic compounds in certain unrefined oils may undergo structural transformations or become more available during storage, leading to a delayed onset of oxidation and prolonged induction times in the early storage stages. Additionally, the formation of secondary antioxidant compounds during storage, for example, through the transformation of phenolics or Millard-type reactions between minor constituents, has also been reported in the literature for specialty oils rich in phenolic compounds. Such processes may induce structural rearrangements or concentration changes in minor components, thereby enhancing the overall antioxidant capacity of the oil [39,68,69]. However, this effect is usually temporary, as prolonged storage ultimately leads to the depletion of antioxidant compounds and accelerated oxidation.

While numerous naturally occurring antioxidants and prooxidants undoubtedly modulate the oxidative behavior of RO, HO, and their blends, a comprehensive characterization of these constituents—encompassing tocopherols, phenolic compounds, carotenoids, pigments, phospholipids, and metal traces—requires a dedicated analytical framework extending beyond the scope of the present study. Importantly, the oxidative responses observed here (PV progression and PDSC induction times) remain consistent with the well-established FA-driven mechanism of oxidation in mixed oils, indicating that the selected analytical endpoints are sufficient to reliably capture the functional behavior of the blends under tested storage conditions.

To contextualize the oxidative findings with other thermal properties, it is worth noting that the thermal behavior of edible oils is governed not only by oxidation kinetics but also by parameters such as the smoke point, which reflects the onset of thermo-volatilization and thermal decomposition. Oils with higher MUFA content and lower PUFA levels—such as HO and HO-rich blends—typically exhibit higher smoke points and better resistance to thermal degradation [70,71]. Although the smoke point was not directly measured in the present study, its potential relevance to RO–HO blends warrants brief consideration. The smoke point is primarily determined by the content of volatile compounds, free fatty acids, and thermally labile minor constituents. Since HO contains lower PUFA levels and naturally fewer pro-oxidative components compared with RO, HO-rich blends would be expected to show slightly higher smoke points and greater thermal resilience.

#### 3.3.3. Multivariate Analysis of Quality Parameters in Rapeseed–Hazelnut Oil Blends

**Figure 3:**
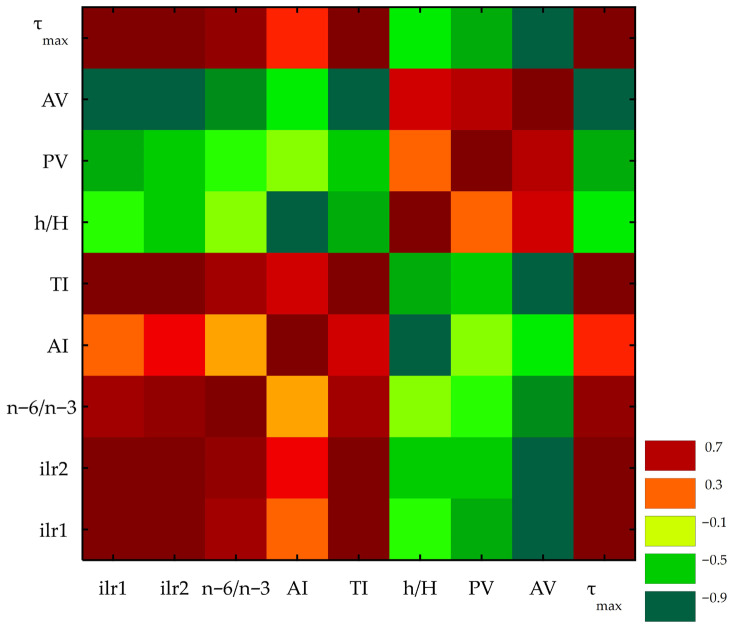
Heatmap of Pearson correlation coefficients (R) computed on ilr-transformed fatty-acid composition together with non-compositional variables across all samples, i.e., the n-6/n-3 ratio, nutritional indices (AI, TI, h/H), and oil stability parameters (PV, AV, τ_max_). Abbreviations: RO, rapeseed oil; HO, hazelnut oil; 3RO:1HO, 1RO:1HO and 1RO:3HO—3:1, 1:1 and 1:3 (*v*/*v*) rapeseed oil to hazelnut oil blends, respectively; n-6/n-3, omega-6/omega-3 ratio; AI, atherogenic index; TI, thrombogenic index; h/H, hypocholesterolemic/hypercholesterolemic index; ilr, isometric log-ratio; 2m, 4m—2 or 4 months of storage; 4 °C and 20 °C—storage temperature.

In addition to the experimental results, multivariate statistical analyses—correlation heatmaps, hierarchical clustering (HCA), and principal component analysis (PCA)—were used to compare the blends, storage conditions, and oxidative behavior. The correlation heatmap (Figure 3) showed a coherent structure dominated by composition.

The MUFA + SFA vs. PUFA contrast (ilr2) showed a strong positive correlation with oxidative stability (τ_max_, R ≈ 0.975) and a strong negative correlation with oxidation products (AV, R ≈ −0.986; PV, R ≈ −0.58), indicating better stability as the composition shifted toward HO. The MUFA vs. SFA contrast (ilr1) showed a similar trend but with slightly weaker correlations (e.g., τ_max_, R ≈ 0.945; AV, R ≈ −0.970). Nutritional indices behaved differently: AI was opposed to h/H (R ≈ −0.99), while TI followed the HO trend (R ≈ 0.99 with ilr2), suggesting a modest increase in SFA with MUFA within these blends.

**Figure 4:**
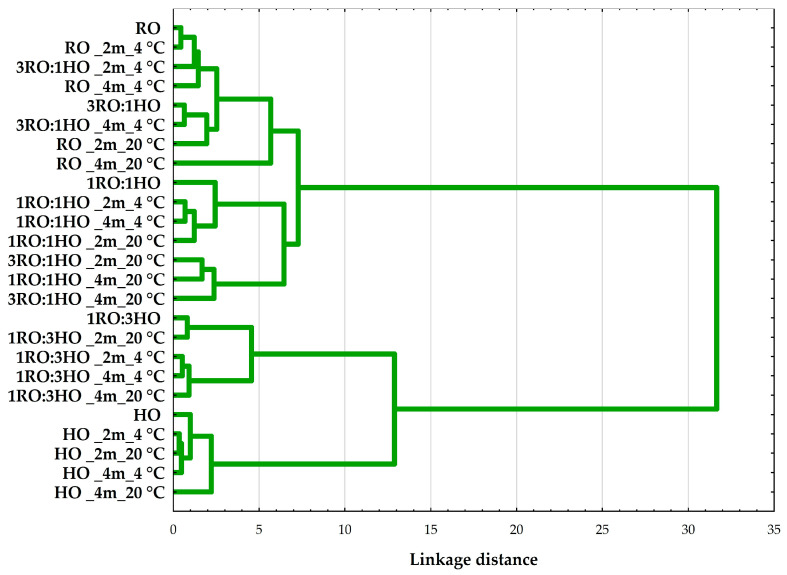
Hierarchical cluster analysis (HCA) dendrogram of rapeseed oil (RO), hazelnut oil (HO), and their blends (3RO:1HO, 1RO:1HO, 1RO:3HO) stored for 2 or 4 months at 4 °C or 20 °C. Ward’s linkage and Euclidean distances were calculated from the full dataset, including standardized ilr1, ilr2 (isometric log-ratio), the n-6/n-3 ratio, nutritional indices (AI, TI, h/H), and oil stability parameters (PV, AV, τ_max_). Labels indicate sample type, storage duration (2m or 4m), and temperature (4 °C or 20 °C).

Consistent with the heatmap, HCA (Figure 4) grouped samples primarily by formulation. HO clustered together with the 1RO:3HO blends, clearly separated from the RO and 3RO:1HO samples. The 1RO:1HO blends occupied an intermediate position, bridging the two groups. Within each formulation group, temperature was the next main factor influencing similarity (20 °C samples clustered separately from 4 °C), and storage time (2 months vs. 4 months) resulted in the smallest endpoint splits. The long linkage connecting the HO/1RO:3HO group to the RO/3RO:1HO group reflects the compositional differences captured by ilr2 and mirrors the stability variations (higher τ_max_, lower AV/PV on the HO-rich side).

PCA (Figure S1) offered a quantitative, low-dimensional view of these patterns. PC1 represented the composition axis (RO ↔ HO), with strong positive loadings for ilr1 (0.932) and ilr2 (0.989), as well as positive contributions from τ_max_ (0.971), TI (0.991), and n-6/n-3 (0.800). Conversely, AV (−0.984) and PV (−0.656) had negative loadings, and h/H also had a negative loading (−0.599). PC2 contrasted AI (−0.852) with h/H (0.796), highlighting secondary nutritional differences within formulations. Sample scores reflected composition: HO was associated with high-positive PC1 values, RO with negative PC1 values, and blends fell along the continuum based on the RO:HO ratio (1RO:3HO positive, 1RO:1HO near the origin, 3RO:1HO negative). Overall, the multivariate analysis consistently shows that composition—specifically the MUFA/PUFA ratio—is the main factor influencing oxidative behavior, while temperature and time have secondary, directional effects.

The findings obtained for RO–HO blends have broader implications for the formulation of mixed plant oils. The observed trends—namely, the modulation of oxidative stability through the relative proportions of MUFA- and PUFA-rich oils, the temperature-dependent progression of peroxide formation, and the blend-specific susceptibility to hydrolytic changes—reflect general mechanisms governing the behavior of edible oils during storage. Therefore, the blending strategy applied here can be extended to other plant oils with complementary lipid characteristics.

For example, oils rich in PUFAs but nutritionally desirable (e.g., flaxseed, hempseed, walnut, or pumpkin seed oil) could be combined with more oxidation-resistant MUFA-dominant oils (e.g., olive, high-oleic sunflower, almond, or hazelnut oil) to achieve enhanced stability while maintaining beneficial FA profiles. Similarly, oils with inherently low resistance to hydrolysis may benefit from combination with oils showing minimal changes in AV under storage, as observed for HO in the present study. In addition to oxidative and hydrolytic stability, oil color is a relevant quality indicator because visible darkening or shifts in hue may accompany chemical deterioration. In edible oils, color changes can reflect not only the formation of secondary oxidation and polymerization products, but also hydrolytic processes leading to increased levels of free FAs and the release or redistribution of endogenous pigments and colloidal material [72]. In cold-pressed oils, the progression of hydrolytic changes (as captured by AV) may therefore be associated with modifications in turbidity and pigment state, which can manifest as gradual changes in perceived color.

Such an approach does not require chemical modification and allows for the design of tailored oil blends optimized for specific storage conditions (e.g., refrigeration vs. room temperature) and intended applications (e.g., culinary, cold-use products, or functional blends). While the exact behavior of individual oil combinations will depend on their unique FA composition and minor constituents, the patterns demonstrated by RO–HO mixtures provide a mechanistic basis for predicting how other binary blends may perform under similar low-temperature storage conditions.

## 4. Conclusions

The present study shows that blending rapeseed oil with hazelnut oil offers an effective way to tailor both the FA profile and the storage stability of edible oils. Increasing the proportion of RO improved nutritional attributes, particularly the n-6/n-3 ratio and h/H index, whereas higher levels of HO enhanced oxidative resistance throughout storage. These patterns highlight a clear interplay between nutritional benefits and technological stability, with neither oil providing an optimal profile on its own. Among the tested formulations, the 1RO:3HO blend represented the most balanced compromise between desirable nutritional indices and resistance to deterioration during storage.

From an application standpoint, these results indicate that rapeseed–hazelnut blends can be formulated to meet specific functional requirements in retail oils, table oils, and culinary fats. RO-leaning blends may be suitable for products emphasizing cardioprotective nutritional characteristics, whereas HO-rich blends offer improved stability under ambient storage and light-heat culinary conditions. Such flexibility makes these blends relevant for industrial development of consumer-ready oils with enhanced functional quality. Future work should include quantitative assessment of minor constituents and their contribution to oxidation pathways, which will support further optimization of blends and facilitate the development of health-oriented, shelf-stable oil formulations.

## Figures and Tables

**Table 1:** Health indices of tested oils and their blends immediately after opening and after two and four months of storage at 4 °C and 20 °C.

Sample	Storage (Month)	Temperature (°C)	n-6/n-3	AI	TI	h/H
RO	0	-	2.09	0.06	0.12	16.45
	2	4	2.06	0.06	0.12	16.74
		20	2.07	0.06	0.12	16.09
	4	4	2.04	0.06	0.12	17.38
		20	2.04	0.06	0.12	16.90
3RO:1HO	0	-	2.23	0.06	0.13	16.30
	2	4	2.22	0.06	0.12	16.82
		20	2.26	0.07	0.13	15.22
	4	4	2.28	0.06	0.13	15.99
		20	2.34	0.07	0.14	14.88
1RO:1HO	0	-	2.71	0.06	0.13	17.27
	2	4	2.77	0.06	0.14	16.06
		20	2.76	0.07	0.14	15.58
	4	4	2.76	0.06	0.14	16.47
		20	2.83	0.07	0.15	14.77
1RO:3HO	0	-	4.51	0.07	0.17	14.39
	2	4	4.36	0.07	0.17	15.16
		20	4.27	0.07	0.17	13.97
	4	4	4.39	0.07	0.17	15.42
		20	4.32	0.06	0.16	15.77
HO	0	-	56.41	0.06	0.18	15.91
	2	4	61.06	0.07	0.19	15.51
		20	60.44	0.07	0.19	15.44
	4	4	65.00	0.07	0.19	15.62
		20	65.47	0.07	0.19	14.75

RO, rapeseed oil; HO, hazelnut oil; 3RO:1HO, 1RO:1HO, and 1RO:3HO—3:1, 1:1, and 1:3 (*v*/*v*) rapeseed oil to hazelnut oil blends, respectively. n-6/n-3, ratio of omega-6 to omega-3 fatty acids; AI, atherogenic index; TI, thrombogenic index; h/H, hypocholesterolemic/hypercholesteremic index. Health indices calculated based on the mean fatty acid composition (n = 3). The detailed FA data used for these calculations are presented in the Supplementary Materials.

**Table 2:** Average acid (AV) and peroxide values (PV) of tested oils and their mixtures immediately after opening and after two and four months of storage at 4 °C and 20 °C.

Sample	Storage (Month)	Temperature (°C)	AV (mg KOH/g Oil)	PV (meq O_2_/kg Oil)
RO	0	-	5.81 ^b^ ± 0.01	6.21 ^e^ ± 0.14
	2	4	5.86 ^b^ ± 0.05	6.54 ^e^ ± 0.28
		20	5.99 ^ab^ ± 0.04	11.06 ^c^ ± 0.95
	4	4	5.90 ^b^ ± 0.06	8.46 ^d^ ± 0.21
		20	6.21 ^a^ ± 0.27	23.72 ^a^ ± 0.22
3RO:1HO	0	-	4.98 ^d^ ± 0.21	4.84 ^fg^ ± 0.05
	2	4	5.09 ^cd^ ± 0.08	5.02 ^f^ ± 0.14
		20	5.16 ^cd^ ± 0.21	5.93 ^e^ ± 0.05
	4	4	5.20 ^cd^ ± 0.03	6.31 ^e^ ± 0.62
		20	5.29 ^c^ ± 0.11	14.61 ^b^ ± 0.57
1RO:1HO	0	-	4.00 ^f^ ± 0.01	3.85 ^h^ ± 0.14
	2	4	4.23 ^ef^ ± 0.20	4.04 ^gh^ ± 0.16
		20	4.24 ^ef^ ± 0.07	4.71 ^fg^ ± 0.60
	4	4	4.22 ^f^ ± 0.07	4.86 ^fg^ ± 0.06
		20	4.48 ^e^ ± 0.24	6.44 ^e^ ± 0.13
1RO:3HO	0	-	3.17 ^g^ ± 0.01	2.54 ^kl^ ± 0.11
	2	4	3.23 ^g^ ± 0.04	2.62 ^jkl^ ± 0.04
		20	3.25 ^g^ ± 0.04	3.56 ^hi^ ± 0.56
	4	4	3.24 ^g^ ± 0.03	3.27 ^hijk^ ± 0.69
		20	3.30 ^g^ ± 0.06	3.71 ^hi^ ± 0.28
HO	0	-	1.98 ^i^ ± 0.01	2.28 ^l^ ± 0.58
	2	4	1.98 ^i^ ± 0.01	2.34 ^l^ ± 0.08
		20	2.16 ^hi^ ± 0.01	3.45 ^hij^ ± 0.08
	4	4	2.00 ^i^ ± 0.05	2.88 ^ijkl^ ± 0.16
		20	2.31 ^h^ ± 0.09	3.56 ^hi^ ± 0.21

RO, rapeseed oil; HO, hazelnut oil; 3RO:1HO, 1RO:1HO, and 1RO:3HO—3:1, 1:1, and 1:3 (*v*/*v*) rapeseed oil to hazelnut oil blends, respectively. The different superscript letters indicate significantly different values (*p* ≤ 0.05). Data are presented as mean values (n = 3) followed by standard deviation (±SD).

**Table 3:** PDSC oxidation time (τ_max_) of tested oils and their blends at 120 °C (immediately after opening and after two and four months of storage at 4 °C and 20 °C).

Sample	Storage (Month)	Temperature (°C)	τ_max_ (min)
RO	0	-	66.28 ^jk^ ± 2.64
	2	4	62.61 ^kl^ ± 2.22
		20	51.58 ^n^ ± 1.32
	4	4	60.41 ^lm^ ± 0.45
		20	35.99 ^p^ ± 0.01
3RO:1HO	0	-	69.32 ^j^ ± 0.83
	2	4	64.02 ^kl^ ± 0.23
		20	56.89 ^m^ ± 3.63
	4	4	74.95 ^i^ ± 4.72
		20	45.48 ^o^ ± 2.67
1RO:1HO	0	-	84.05 ^h^ ± 0.11
	2	4	82.87 ^h^ ± 0.75
		20	81.55 ^h^ ± 1.73
	4	4	89.54 ^g^ ± 0.18
		20	79.64 ^hi^ ± 1.13
1RO:3HO	0	-	115.31 ^ef^ ± 1.10
	2	4	118.16 ^de^ ± 0.91
		20	116.17 ^def^ ± 2.17
	4	4	120.85 ^d^ ± 1.63
		20	111.86 ^f^ ± 2.35
HO	0	-	160.32 ^c^ ± 3.84
	2	4	167.02 ^b^ ± 1.07
		20	164.68 ^bc^ ± 1.73
	4	4	177.07 ^a^ ± 2.23
		20	174.99 ^a^ ± 3.44

RO, rapeseed oil; HO, hazelnut oil; 3RO:1HO, 1RO:1HO, and 1RO:3HO—3:1, 1:1, and 1:3 (*v*/*v*) rapeseed oil to hazelnut oil blends, respectively. Determined data are presented as mean values (n = 3) followed by standard deviation (±SD). The different superscript letters indicate significantly different values (*p* ≤ 0.05).

## Data Availability

The original contributions presented in the study are included in the article/Supplementary Material, further inquiries can be directed to the corresponding author.

## Supplementary Materials

The following supporting information can be downloaded at https://www.mdpi.com/article/10.3390/foods14234008/s1, Table S1: Fatty acid composition (%) of tested oils and their blends immediately after opening and after two and four months of storage at 20°C, Table S2: Fatty acid composition (%) of tested oils and their blends after two and four months of storage at 4°C, Figure S1: Principal component analysis (PCA) of rapeseed oil (RO), hazelnut oil (HO), and their blends (3RO:1HO, 1RO:1HO, 1RO:3HO) stored for 2 or 4 months at 4 °C or 20 °C. (a) Scores plot showing sample distribution on PC1 (71.51%) and PC2 (18.13%). (b) Loading plot for PC1 vs. PC2. Labels encode formulation, storage time (2m or 4m), and temperature (4 °C or 20 °C).
