# Supplementary material for: Toward Functional Oil Blends: Physicochemical and Nutritional Evaluation of Rapeseed–Hazelnut Oil Mixtures
Source: Foods. 2025 Nov 22;14(23):4008. doi: 10.3390/foods14234008 (PMC12691934; doi:10.3390/foods14234008)
Supplement: Supplementary file 1 [file foods-14-04008-s001.zip › foods-3970597-supplementary.docx]

**Table S1.** Fatty acid composition (%) of tested oils and their blends immediately after opening and after two and four months of storage at 20°C.

| Composition after opening (%) | | | | | | Composition after 2 months of storage (%) | | | | | Composition after 4 months of storage (%) | | | | |
| --- | --- | --- | --- | --- | --- | --- | --- | --- | --- | --- | --- | --- | --- | --- | --- |
| Fatty  acid | RO | 3RO:1HO | 1RO:1HO | 1RO:3HO | HO | RO | 3RO:1HO | 1RO:1HO | 1RO:3HO | HO | RO | 3RO:1HO | 1RO:1HO | 1RO:3HO | HO |
| C14:0 | 0.06±0.01 | 0.08±0.01 | 0.06±0.01 | 0.08±0.01 | 0.04±0.01 | 0.10±0.02 | 0.09±0.01 | 0.06±0.01 | 0.07±0.01 | 0.04±0.01 | 0.08±0.01 | 0.07±0.01 | 0.11±0.01 | 0.06±0.01 | 0.05±0.01 |
| C16:0 | 5.38±0.96 | 5.42±0.01 | 5.17±0.01 | 6.19±0.43 | 5.67±0.31 | 5.46±1.41 | 5.78±0.01 | 5.71±0.07 | 6.35±0.33 | 5.82±0.19 | 5.21±0.07 | 5.92±0.10 | 5.96±0.70 | 5.66±0.12 | 6.08±0.28 |
| C16:1 | 0.25±0.04 | 0.28±0.01 | 0.24±0.01 | 0.24±0.01 | 0.21±0.01 | 0.29±0.01 | 0.29±0.01 | 0.26±0.01 | 0.25±0.01 | 0.21±0.01 | 0.28±0.01 | 0.27±0.01 | 0.30±0.01 | 0.24±0.01 | 0.22±0.01 |
| C17:0 | 0.13±0.01 | 0.12±0.01 | 0.09±0.01 | 0.05±0.01 | 0.06±0.01 | 0.13±0.03 | 0.11±0.01 | 0.09±0.01 | 0.08±0.01 | 0.07±0.01 | 0.12±0.01 | 0.12±0.01 | 0.13±0.01 | 0.08±0.01 | 0.06±0.01 |
| C17:1 | 0.07±0.01 | 0.07±0.01 | 0.08±0.01 | 0.05±0.01 | 0.10±0.01 | 0.06±0.01 | 0.09±0.01 | 0.08±0.01 | 0.09±0.01 | 0.11±0.01 | 0.08±0.01 | 0.08±0.02 | 0.10±0.01 | 0.10±0.01 | 0.10±0.01 |
| C18:0 | 2.7±0.11 | 2.80±0.01 | 2.76±0.01 | 2.68±0.08 | 2.77±0.09 | 2.60±0.24 | 2.68±0.01 | 2.71±0.19 | 2.69±0.03 | 2.89±0.03 | 2.68±0.06 | 2.73±0.02 | 2.61±0.20 | 2.81±0.01 | 2.78±0.05 |
| C18:1 n-9c | 61.55±0.18 | 65.15±0.01 | 70.26±0.05 | 75.17±0.12 | 81.09±0.09 | 61.58±0.30 | 65.12±0.02 | 70.02±0.24 | 74.26±0.07 | 80.66±0.08 | 62.01±0.08 | 65.18±0.03 | 70.35±0.22 | 75.31±0.01 | 80.45±0.06 |
| C18:2 n-6c | 18.91 ±0.11 | 16.90 ±0.01 | 14.66 ±0.06 | 12.32 ±0.25 | 9.59±0.20 | 18.78±0.45 | 16.81±0.08 | 14.61±0.07 | 12.50±0.22 | 9.67±0.18 | 18.40±0.15 | 16.76±0.02 | 14.25±0.07 | 12.09±0.09 | 9.82±0.06 |
| C18:3 n-3 | 8.70±0.08 | 7.34±0.01 | 5.20 ±0.01 | 2.65 ±0.01 | 0.14±0.01 | 8.85±0.04 | 7.14±0.01 | 5.05±0.04 | 2.85±0.01 | 0.14±0.01 | 8.62±0.03 | 6.88±0.04 | 4.88±0.04 | 2.69±0.01 | 0.14±0.01 |
| C20:0 | 0.76±0.03 | 0.64±0.01 | 0.51±0.02 | 0.18±0.01 | 0.15±0.01 | 0.69±0.14 | 0.63±0.02 | 0.45±0.04 | 0.31±0.01 | 0.15±0.01 | 0.83±0.06 | 0.66±0.01 | 0.42±0.04 | 0.34±0.01 | 0.12±0.01 |
| C20:1c | 1.16±0.24 | 0.96±0.01 | 0.77±0.04 | 0.31±0.01 | 0.15±0.01 | 1.22±0.23 | 0.97±0.01 | 0.72±0.10 | 0.47±0.01 | 0.22±0.01 | 1.30±0.02 | 1.04±0.03 | 0.73±0.11 | 0.51±0.01 | 0.17±0.03 |
| C20:3 n-3 | 0.33 ±0.09 | 0.24 ±0.01 | 0.20 ±0.01 | 0.08 ±0.01 | 0.03±0.01 | 0.24±0.12 | 0.29±0.01 | 0.24±0.04 | 0.08±0.01 | 0.02±0.06 | 0.39±0.01 | 0.29±0.01 | 0.16±0.04 | 0.11±0.01 | 0.01±0.02 |
| Σ SFA | 9.03 | 89.06 | 8.59 | 9.18 | 8.69 | 8.98 | 9.29 | 9.02 | 9.50 | 8.97 | 8.92 | 9.50 | 9.23 | 8.95 | 9.09 |
| Σ MUFA | 63.03 | 66.46 | 71.35 | 75.77 | 81.55 | 63.15 | 66.47 | 71.08 | 75.07 | 81.20 | 63.67 | 66.57 | 71.48 | 76.16 | 80.94 |
| Σ PUFA | 27.94 | 24.48 | 20.06 | 15.05 | 9.76 | 27.87 | 24.24 | 19.90 | 15.43 | 9.83 | 27.41 | 23.93 | 19.29 | 14.89 | 9.97 |

**Table S2.** Fatty acid composition (%) of tested oils and their blends after two and four months of storage at 4°C.

| Composition after 2 months of storage (%) | | | | | | Composition after 4 months of storage (%) | | | | |
| --- | --- | --- | --- | --- | --- | --- | --- | --- | --- | --- |
| Fatty  acid | RO | 3RO:1HO | 1RO:1HO | 1RO:3HO | HO | RO | 3RO:1HO | 1RO:1HO | 1RO:3HO | HO |
| C14:0 | 0.08±0.01 | 0.08±0.01 | 0.06±0.01 | 0.06±0.01 | 0.04±0.01 | 0.09±0.01 | 0.09±0.01 | 0.06±0.01 | 0.05±0.01 | 0.04±0.01 |
| C16:0 | 5.27±0.03 | 5.25±0.04 | 5.54±0.04 | 5.88±0.15 | 5.80±0.16 | 5.06±0.01 | 5.50±0.33 | 5.40±0.40 | 5.79±0.04 | 5.77±0.40 |
| C16:1 | 0.28±0.01 | 0.28±0.01 | 0.25±0.01 | 0.24±0.01 | 0.22±0.01 | 0.28±0.01 | 0.29±0.01 | 0.26±0.01 | 0.23±0.01 | 0.20±0.01 |
| C17:0 | 0.13±0.01 | 0.11±0.01 | 0.09±0.01 | 0.07±0.01 | 0.07±0.01 | 0.13±0.01 | 0.12±0.01 | 0.10±0.01 | 0.08±0.01 | 0.07±0.01 |
| C17:1 | 0.07±0.01 | 0.08±0.01 | 0.09±0.01 | 0.08±0.01 | 0.10±0.01 | 0.09±0.01 | 0.09±0.01 | 0.09±0.01 | 0.10±0.01 | 0.10±0.01 |
| C18:0 | 2.64±0.01 | 2.75±0.01 | 2.77±0.01 | 2.77±0.01 | 2.79±0.01 | 2.76±0.01 | 2.75±0.04 | 2.80±0.19 | 2.84±0.01 | 2.78±0.10 |
| C18:1 n-9c | 61.91±0.01 | 65.19±0.01 | 69.96±0.02 | 74.87±0.14 | 80.67±0.05 | 62.05±0.01 | 65.38±0.02 | 70.19±0.14 | 75.25±0.03 | 80.84±0.13 |
| C18:2 n-6c | 18.62±0.01 | 16.85±0.01 | 14.66±0.04 | 12.37±0.08 | 9.77±0.18 | 18.41±0.01 | 16.68±0.02 | 14.47±0.21 | 12.08±0.04 | 9.75±0.15 |
| C18:3 n-3 | 8.72±0.01 | 7.28±0.01 | 5.10±0.02 | 2.73±0.01 | 0.14±0.01 | 8.66±0.01 | 7.02±0.01 | 5.02±0.03 | 2.64±0.01 | 0.14±0.01 |
| C20:0 | 0.76±0.01 | 0.71±0.01 | 0.50±0.01 | 0.33±0.01 | 0.16±0.01 | 0.84±0.01 | 0.70±0.01 | 0.55±0.04 | 0.33±0.01 | 0.14±0.01 |
| C20:1c | 1.19±0.01 | 1.10±0.03 | 0.78±0.06 | 0.49±0.01 | 0.22±0.03 | 1.25±0.01 | 1.08±0.05 | 0.83±0.08 | 0.50±0.01 | 0.16±0.02 |
| C20:3 n-3 | 0.33±0.02 | 0.32±0.01 | 0.20±0.07 | 0.11±0.04 | 0.02±0.01 | 0.38±0.01 | 0.30±0.01 | 0.23±0.02 | 0.11±0.01 | 0.01±0.01 |
| Σ SFA | 8.88 | 8.90 | 8.96 | 9.11 | 8.86 | 8.88 | 9.16 | 8.91 | 9.09 | 8.80 |
| Σ MUFA | 63.45 | 66.65 | 71.08 | 75.68 | 81.21 | 63.67 | 66.84 | 71.37 | 76.08 | 81.30 |
| Σ PUFA | 27.67 | 24.45 | 19.96 | 15.21 | 9.93 | 27.45 | 24.00 | 19.72 | 14.83 | 9.90 |

RO, rapeseed oil; 3RO:1HO; 3:1 (v/v) rapeseed oil to hazelnut oil mixture; 1RO:1HO, 1:1 (v/v) rapeseed oil to hazelnut oil mixture; 1RO:3HO, 1:3 (v/v) rapeseed oil to hazelnut oil mixture; H, hazelnut oil. Data are presented as mean values followed by standard deviation (±SD).

| 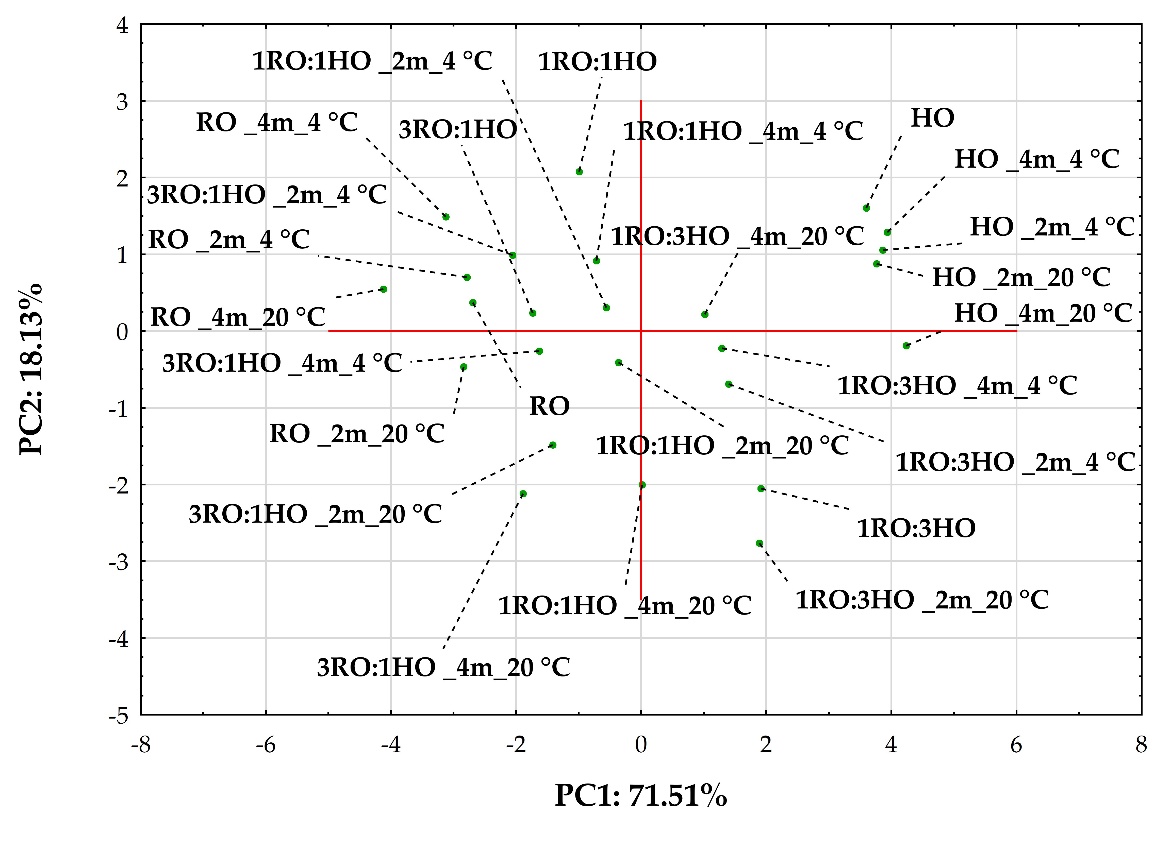 | **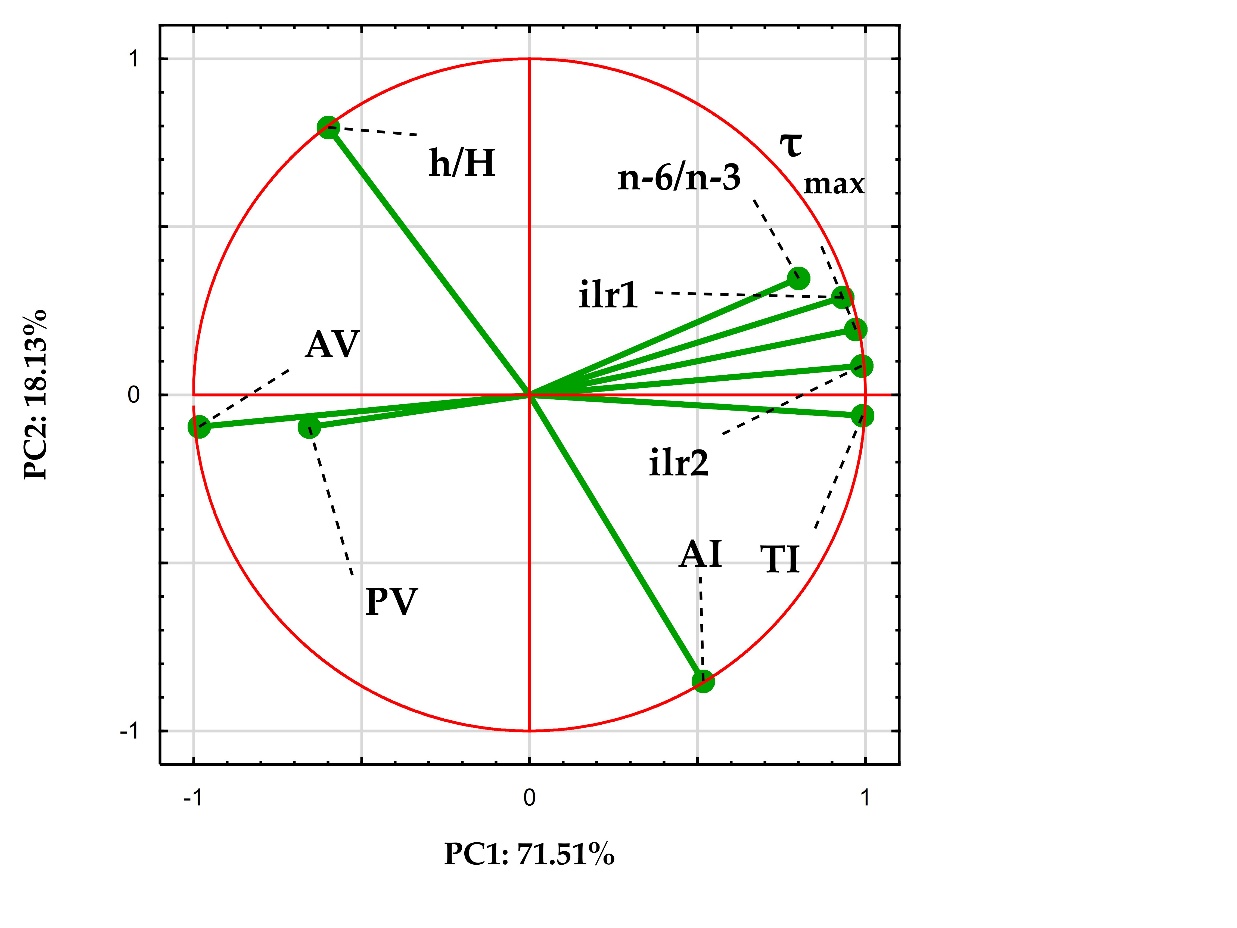** |
| --- | --- |
| (**a**) | (**b**) |

**Figure S1.** Principal component analysis (PCA) of rapeseed oil (RO), hazelnut oil (HO), and their blends (3RO:1HO, 1RO:1HO, 1RO:3HO) stored for 2 or 4 months at 4 °C or 20 °C. (**a**) Scores plot showing sample distribution on PC1 (71.51%) and PC2 (18.13%). (**b**) Loading plot for PC1 vs. PC2. Labels encode formulation, storage time (2m or 4m), and temperature (4 °C or 20 °C).
